# Towards precision medicine in diabetes? A critical review of glucotypes

**DOI:** 10.1371/journal.pbio.3000890

**Published:** 2021-03-11

**Authors:** Adam Hulman, Yuri D. Foreman, Martijn C. G. J. Brouwers, Abraham A. Kroon, Koen D. Reesink, Pieter C. Dagnelie, Carla J. H. van der Kallen, Marleen M. J. van Greevenbroek, Kristine Færch, Dorte Vistisen, Marit E. Jørgensen, Coen D. A. Stehouwer, Daniel R. Witte

**Affiliations:** 1 Steno Diabetes Center Aarhus, Aarhus University Hospital, Aarhus N, Denmark; 2 Department of Internal Medicine, Maastricht University Medical Center+, Maastricht, the Netherlands; 3 CARIM School for Cardiovascular Diseases, Maastricht University, Maastricht, the Netherlands; 4 Steno Diabetes Center Copenhagen, Gentofte, Denmark; 5 Department of Biomedical Sciences, University of Copenhagen, Copenhagen, Denmark; 6 National Institute of Public Health, Southern Denmark University, Copenhagen, Denmark; 7 Aarhus University, Aarhus C, Denmark; Duke University, UNITED STATES

## Abstract

In response to a study previously published in PLOS Biology, this Formal Comment thoroughly examines the concept of ’glucotypes’ with regard to its generalisability, interpretability and relationship to more traditional measures used to describe data from continuous glucose monitoring.

Although the promise of precision medicine has led to advances in the recognition and treatment of rare monogenic forms of diabetes, its impact on prevention and treatment of more common forms of diabetes has been underwhelming [1]. Several approaches to the subclassification of individuals with, or at high risk of, type 2 diabetes have been published recently [2–4]. Hall and colleagues introduced the concept of “glucotypes” in a research article [3] that has received enormous attention in the highest impact scientific journals [5–8], mostly in relation to precision medicine. The authors developed an algorithm to identify patterns of glucose fluctuations based on continuous glucose monitoring (CGM). They named the 3 identified patterns: “low variability,” “moderate variability,” and “severe variability” glucotypes. Each individual was characterised by the proportion of time spent in the 3 glucotypes and was assigned to an overall glucotype based on the highest proportion. They argued that glucotypes provide the advantage of taking into account a more detailed picture of glucose dynamics, in contrast to commonly used single time point or average-based measures, revealing subphenotypes within traditional diagnostic categories of glucose regulation. Even though the study was based on data from only 57 individuals without a prior diabetes diagnosis, others have interpreted the results as indicating that glucotypes might identify individuals at an early stage of glucose dysregulation, suggesting a potential role in diabetes risk stratification and prevention [5]. However, before glucotypes can become “an important tool in early identification of those at risk for type 2 diabetes” [3], the concept requires thorough validation. Therefore, we explore the generalisability and interpretability of glucotypes and their relationship to traditional CGM-based measures.

We used data from The Maastricht Study [9] and the PRE-D Trial [10] comprising a total number of 770 diabetes-free individuals with a 7-day CGM registration. We observed that the average proportion of time spent in the low variability glucotype was low both in The Maastricht Study (6%) and the PRE-D Trial (4%), compared to 20% in the original study. A reason for the difference may be that our study populations were on average 11 to 12 years older and that the PRE-D Trial (*n* = 116) included only overweight and obese individuals with prediabetes. In The Maastricht Study, the median (Q1 to Q3) body mass index was 25.9 kg/m^2^ (23.4 to 28.7), and 72% had normal glucose tolerance. As a logical consequence, the severe glucotype was most common in the PRE-D Trial (55%). Regardless, our data show that the initial estimates of the different glucotype prevalences do not necessarily generalise to other populations, especially in age groups at increased risk of type 2 diabetes.

Hall and colleagues described glucotypes as a new measure of glucose variability, a clinically relevant metric of glycaemic patterns [3]. In the figures accompanying the original publication, the low variability pattern was characterised by both the lowest mean glucose level and variation, while the severe pattern had both the highest mean glucose level and variation. As such, these examples did not give an intuition whether glucotypes were predominantly driven by glucose variability or by mean glucose levels. We therefore present 3 examples from the PRE-D Trial (Fig 1). The first 2 profiles are very similar with regard to glucose variability. Thus, the driver of the most severe glucotype of the second participant is clearly the slightly higher mean glycaemic level. Also, even though the third participant has a much larger variation than the first two, the proportion of time in the severe glucotype is not higher than for the second participant as one would expect from a classical measure of glucose variability. To investigate this further, we assessed the association between glucotypes and classical CGM measures, i.e., the mean CGM glucose level (Fig 2A) and the coefficient of variation (Fig 2B) in The Maastricht Study. The scatterplots show a clear association between the mean CGM glucose and glucotypes. They also suggest that participants with a high proportion of time in the moderate glucotype do not have high variation in glucose. Rather than a biological feature, this may well be a methodological consequence of being assigned to the middle cluster. If large fluctuations were present, glucose levels would reach either low or high values, resulting in a higher proportion of time spent in the low or severe glucotypes, respectively (assuming a strong association between glucotypes and mean CGM glucose). Therefore, we decided to quantify this association using regression analysis where glucotype proportions were the outcomes, and the mean CGM glucose concentration was the independent variable modelled with natural cubic splines (more details on the specification of the models are given in Supporting information S1–S3 Codes). Then, we used the equation estimated in The Maastricht Study to predict glucotypes in the external validation sample (PRE-D Trial, Fig 2C). First, similarly to Hall and colleagues, we assigned individuals to the pattern with the highest proportion of time and then compared the predicted and the observed glucotypes. We found that in 107 out of 116 individuals, the glucotype was predicted correctly when using only the mean CGM glucose value. When considering the glucotypes as continuous proportions of time, the root mean squared errors (RMSEs) were 0.05, 0.09, and 0.07 for the low, moderate, and severe variability glucotypes, respectively, indicating good predictive ability. These results demonstrate that glucotypes either mainly reflect the mean CGM glucose level or do not translate to external datasets (e.g., due to overfitting). To investigate this further, we conducted the same analyses as described for the PRE-D Trial in the original data from Hall and colleagues and found a slightly weaker, but still strong association between mean CGM glucose levels and glucotypes. Using the regression model from The Maastricht Study, we could correctly predict 79% of the glucotypes, while the RMSEs were 0.11, 0.15, and 0.13.

**Fig 1:**
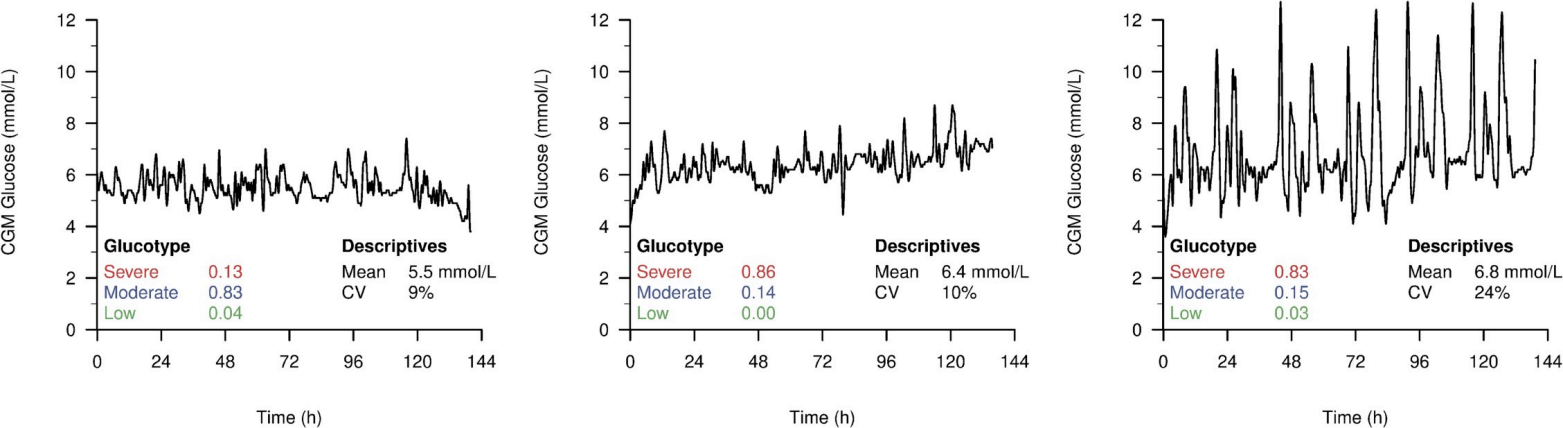
Example CGM profiles of participants in the PRE-D Trial with corresponding proportion of time spent in different glucotypes and conventional measures (mean and CV). CGM, continuous glucose monitoring; CV, coefficient of variation.

**Fig 2:**
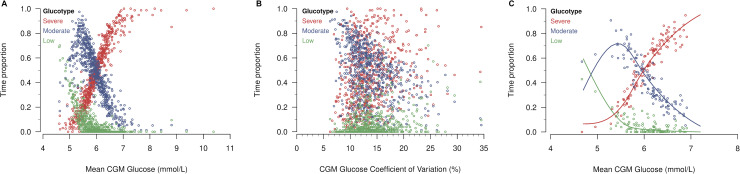
Observed proportion of time spent in the 3 glucotypes by mean CGM glucose (A) and coefficient of variation (B) in The Maastricht Study, and by mean CGM glucose in the PRE-D Trial (C) alongside predicted proportions based on the regression analysis in The Maastricht Study. CGM, continuous glucose monitoring.

Although the transformation of continuous measures into categorical ones is a common procedure in clinical research, assigning individuals to the glucotype with the highest proportion of time runs very much against the “precision” tenet of precision medicine. In line with this, a recent study has demonstrated how simple clinical features outperformed clusters in predicting relevant clinical outcomes [11]. This is especially problematic when a method does not provide clear separation between clusters, which can be quantified by calculating relative entropy [12]. A relative entropy of zero would mean that all individuals spend one-third of the time in each of the 3 glucotypes, while a value of one would indicate that each individual spends the entire time period in only one of the 3 glucotypes. In the original cohort of Hall and colleagues [3], we calculated a relative entropy of 0.24 indicating that cluster separation is far from optimal and together with the previous results question the claim that the glucotype is really a “more comprehensive measure of the pattern of glucose excursions than the standard laboratory tests in current use” [3].

In conclusion, we demonstrate in 2 large, external datasets, that the assessment of glucotypes does not offer more novel insights than the mean CGM glucose, highlighting the importance of large development datasets and external validation for data-driven algorithms. As CGM is becoming more widely used in large clinical studies also among individuals without diabetes, glucose patterns derived from CGMs will be an important focus area in future diabetes research. However, it is important that scientific scrutiny precedes the introduction of emerging tools with a promise of identifying individuals at high risk of type 2 diabetes and its late complications at an earlier stage of disease progression, especially in an observational setting. Furthermore, future efforts towards precision medicine for diabetes prevention and treatment should go beyond the glucocentric approach we have seen so far. We know that hyperglycaemia is a late feature of diabetes development and that patients benefit most from a multifactorial treatment approach [13]. A multifactorial approach, with relevance to the aetiology of micro- and macrovascular complications, may also yield a more clinically useful risk stratification of nondiabetic individuals [14]. Even so, if we aim for precision medicine, we should aim to retain as much precision as possible at every step of the process, by treating determinants and outcomes as continuous measures if possible and by retaining information on the uncertainty of any hard classification such as cluster membership.

## Supporting information

S1 Code This R script demonstrates the assessment of glucotypes in the PRE-D Trial. It uses files from Alessandra Breschi’s GitHub page (https://github.com/abreschi/shinySpecClust). These files were accessed and downloaded on of July 5, 2019. Glucotypes in The Maastricht Study were assessed with the same method (code not shown). The script also includes the code to create Fig 1 displaying individual glucose trajectories. To avoid plotting individual datapoints, we calculate a 10-minute moving average for the glucose values.
(R) Click here for additional data file.

S2 Code The regression model is fitted with glucotypes as outcomes and mean CGM glucose exposure. R code is also available for Fig 2 showing the association between glucotypes and mean CGM glucose (Fig 2A) and the coefficient of variation (Fig 2B). CGM, continuous glucose monitoring.
(R) Click here for additional data file.

S3 Code We are using the regression model developed in The Maastricht cohort (see S2 Code) to predict glucotypes based on mean CGM glucose in the PRE-D Trial. Then, the RMSE and the number of correctly classified individuals are calculated. Also, relative entropy is calculated in the Stanford cohort from the original paper [3]. CGM, continuous glucose monitoring; RMSE, root mean squared error.
(R) Click here for additional data file.
